# Commentary: Comparative Transcriptome Analysis of *Raphanus sativus* Tissues

**DOI:** 10.3389/fpls.2015.01191

**Published:** 2016-01-05

**Authors:** Xiaofeng Gu, Tiegang Lu

**Affiliations:** Biotechnology Research Institute/National Key Facility for Genetic Resources and Gene Improvement, The Chinese Academy of Agricultural SciencesBeijing, China

**Keywords:** *Raphanus sativus*, transcription factors, RNA sequencing (RNA-Seq), trancriptome, simple sequence repeats (SSRs)

Obtaining sequence information for a plant species of interest greatly facilitates functional genomic research. However, many plant species have not been sequenced due to the complexities of their genomes and the high cost of sequencing. Therefore, the transcriptomes of several plants were sequenced using next-generation sequencing technology. RNA-seq technology is an important high-throughput tool for both gene mapping and the transcriptome analysis of non-model organisms lacking genome information (Schuster, 2008; Wang et al., 2009; Klie et al., 2012; Drogue et al., 2014; Druege et al., 2014). Additionally, RNA-seq data can help connect the genome to gene function (Adams, 2008) and have tremendously expanded the volume of transcriptome information available for non-model plants. The uniformly processed and matched nature of the transcriptome data also facilitates their integration with upstream factor-binding and chromatin-modification signals (Gerstein et al., 2014).

The transcriptome sequencing analyses of single tissues from *Raphanus sativus* were recently reported because it is an important *Brassicaceae* plant with both economic value and medicinal properties (Wang et al., 2013; Zhang et al., 2013). These studies have advanced functional genomic investigations of *R. sativus.* However, routine analysis and annotation methods have impeded our understanding of the functional genomics and medicinal potential of *R. sativus.* The traditional analysis of transcriptome data focuses on evaluating the expression changes of single genes. The accumulating volume of transcriptome data requires the development of new methods and tools for data integration. The network theory and similar methods have been widely used to analyze high-throughput data in the field of systems biology (Ashburner, 2000; Lee et al., 2010; Kleessen et al., 2013). Cytoscape is an open source software used for visualizing molecular interaction networks and biological pathways. The software can also integrate these networks with annotation and gene expression data (Shannon et al., 2003; Ma et al., 2014).

Using *R. sativus* as a model, Wu et al. (2015) combined the leaf sequencing data with the root sequencing data and obtained the better transcriptome assembly of *R. sativus*. For example, the authors obtained 68,086 unigenes with an average length of 576 bp by Trinity program, which represents a rich genome resource of *R. sativus*. Moreover, a total of 31,875 potential simple sequence repeats (SSRs) were identified in this article. SSRs are repeating DNA sequences of 2–6 bp and widely used in molecular marker identification, high-throughput genome mapping, and the analysis of species diversity. Thus, the identification of multiple SSRs in *R. sativus* will be very helpful for the functional genomic research of *R. sativus* in the future. The authors (Wu et al., 2015) used Cytoscape software to annotate these differentially expressed genes (Figure 1). The annotations effectively explained the GO classifications for the differentially expressed genes found in leaf and root tissues and are very important for functional genomics research of *R. sativus*. In addition, Wu et al. examined the TFs (transcription factors) in *R. sativus* and constructed a TF-based regulation network using Cytoscape software to elucidate the regulatory network controlling the development of *R. sativus*. This analysis may provide critical insights into the regulatory role of TFs in the development of *R. sativus*.

**Figure 1:**
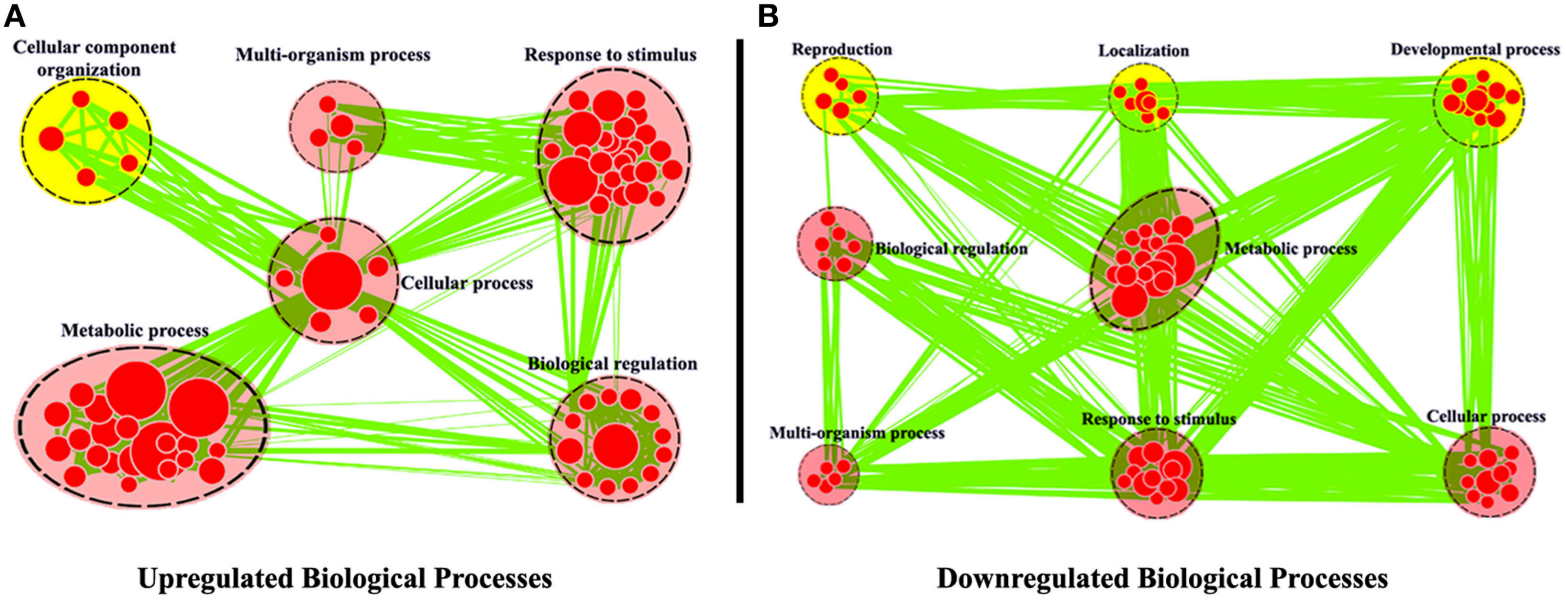
**Biological process analysis of differentially expressed genes between leaf and root tissues**. GO modules enriched with up-regulated DEGs **(A)** and down-regulated DEGs **(B)** were visualized by the EnrichmentMap in Cytoscape. The red and yellow circles indicate the common and different biological processes between up-regulated and down-regulated DEGs, respectively (From the article Wu et al., 2015).

This study is not only an important genomic resource for *R. sativus* because it provides both transcriptome sequencing and a TF-based interaction network but also will also facilitate future network-based functional genomic analyses and will provide insights into the systematic analysis of high-throughput sequencing data.

## Conflict of interest statement

The authors declare that the research was conducted in the absence of any commercial or financial relationships that could be construed as a potential conflict of interest.
